# Determining the cytotoxicity of the Minimum Inhibitory Concentration (MIC) of silver and zinc oxide nanoparticles in ESBL and carbapenemase producing *Proteus mirabilis* isolated from clinical samples in Shiraz, Southwest Iran

**DOI:** 10.1186/s13104-023-06402-2

**Published:** 2024-01-29

**Authors:** Farshad Kakian, Esmaeil Mirzaei, Afagh Moattari, Sara Takallu, Abdollah Bazargani

**Affiliations:** 1 Department of Bacteriology and Virology, School of Medicine, Student Research Committee of Shiraz, University of Medical Sciences, Shiraz, Iran; 2 Department of Medical Nanotechnology, School of Advanced Medical Sciences and Technologies, Shiraz University of Medical Sciences, Shiraz, Iran; 3 Department of Bacteriology and Virology, School of Medicine, Shiraz University of Medical Science, Shiraz, Iran

**Keywords:** *Proteus mirabilis*, Drug Resistance, Silver, Zinc oxide, Cell Culture techniques

## Abstract

**Objective:**

*Proteus mirabilis* is related to serious infections. The present study was designed to investigate the minimum inhibitory concentration (MIC) of silver nanoparticles (AgNPs) and zinc oxide nanoparticles (ZnONPs) and cytotoxicity among *P. mirabilis* isolates recovered from clinical samples in Shiraz.

**Results:**

A total of 100 *P. mirabilis* isolates were screened by biochemical tests and polymerase chain reaction (PCR). Also, 25 (25%) and 7 (7%) isolates were positive for extended-spectrum beta-lactamase (ESBLs) and carbapenemase, respectively. Synthesized nanoparticles were characterized by UV–vis spectrum, X-ray diffraction (XRD), and electron microscopy. The average size of AgNPs and ZnONPs in the present study is 48 and < 70 nm, respectively. The MIC and the MBC of the ZnONPs were in the range of 31.25 µg/ml and 62.5 µg/mL, respectively. Also, for AgNPs, the MIC and the MBC were in the range of 7.8 µg/mL and 15.6 µg/mL, respectively. MTT (3-(4,5-Dimethylthiazol-2-yl)-2,5-diphenyltetrazolium bromide) assay in a primary culture of fibroblast L929 cells for this MIC indicated biocompatibility and low cytotoxicity of Ag NPs and for ZnONPs indicated significant cytotoxicity. Also, a MIC of AgNPs can be used as a therapeutic concentration without the effect of cytotoxicity in human cells.

## Introduction

*Proteus mirabilis* has different mechanisms of antibiotic resistance [1, 2]. Among these mechanisms, ESBLs and carbapenemase enzymes can be mentioned [3, 4]. These resistant strains lead to increased mortality, treatment costs, and increase the length of hospitalization [5, 6]. Nowadays, a lot of attention has been paid to the science of nanotechnology to develop antimicrobial drugs. Among AgNPs and ZnONPs have been successful in this direction due to their strong antimicrobial properties [7]. The antimicrobial mechanisms of nanoparticles are not fully understood, but accepted mechanisms include oxidative stress induction, metal ion release, non-oxidative mechanisms, cell membrane damage, DNA damage, and free radical generation [8]. Also, the antimicrobial properties of nanoparticles depend on different factors such as their synthesis method, nanoparticle size, morphology, etc. [9]. However, the number of studies that have simultaneously investigated the antibacterial and cytotoxicity effects of AgNPs and ZnONPs on *P. mirabilis* isolated from clinical samples is few. Therefore, the present study was designed with these goals.

## Main text

## Materials and methods

### Samples and bacterial isolates

This cross-sectional study was conducted in the bacteriology and virology department of Shiraz University of Medical Sciences. From September 2021 to April 2022, a total of 100 isolates of *P. mirabilis* were collected from clinical samples (blood, urine, wound, sputum, etc.) of outpatients and inpatients attending university hospitals (Namazi, Faghihi, Hafez, Amir, Ebnesina) in Shiraz.

### Characterization assays

The isolates were recognized using Gram staining, biochemical tests (Urea, Sulfide Indole Motility Medium, Triple Sugar Iron, Methyl Red / Voges-Proskauer, Simon citrate, etc. of Merck Co., Germany), and PCR with the *ure R* primer [10].

### Detection of ESBLs and carbapenemase

The production of ESBLs was investigated according to Clinical and Laboratory Standards Institute (CLSI) guidelines in isolates resistant to ceftazidime, cefotaxime, or ceftriaxone by a phenotypic confirmatory test. If the diameter of halo ceftazidime/clavulanic acid was greater than 5 mm in comparison to ceftazidime, ESBL production was confirmed [11].

The modified Inactivation Method (mCIM) test is done in isolates resistant to imipenem or meropenem. For mCIM one loopful (1-µL) of the isolate suspension was inoculated in a tube containing 2 ml of TSB. A meropenem (MEM) disk is submerged in a tube, and the tube is incubated at 35 °C for 4 h ± 15 min. The disk is then removed from the tube and placed on an MH agar (Merck Co., Germany) plate upon which *E. coli* ATCC 25,922 (carbapenem-susceptible) has been newly applied. The plate is incubated at 35 °C for 16 to 20 h. Finally, the halo diameter is checked [12].

### AgNPs and ZnONPs

A chemical reduction method was used to synthesize AgNPs. Ascorbic acid was used as a reducing agent and sodium citrate as a stabilizing agent. First, ascorbic acid was dissolved in 64 ml of distilled water and then 49.52 mg of sodium citrate was added to it. Then we adjusted the pH to 10.5 with NaOH. In the next step, we added silver salt (AgNo3) dissolved in distilled water (10 mg in 64 ul) to the solution. We allowed AgNPs to form on the hot plate for 30 min at a temperature of 30 ^C^. In the last step, centrifugation was performed at 10,000 rpm for 40 min and falcon sediment was used [13].

The AgNPs were characterized by Unico UV-2100 Spectrophotometer. The scanning range for the samples was 300–500 nm. The double distilled water was used as a blank reference. Also, the X-ray diffraction data were obtained by X-ray diffractometer (ASENWARE, AW-XDM300 with Cu Kα radiation sources (λ = 0.154 nm) by operating voltage 18 kV and the range of diffraction angle were set as 10°-80°). The morphology and size of the particle were determined using a scanning electron microscope (SEM), (X13, Philips, America Leo 1430vp) [14].

In this study, ZnONPs powder was purchased from the Burhan nanoscale innovators company of the Ferdowsi University of Mashhad and used. These nanoparticles have been characterized using UV-Vis, XRD, and transmission electron microscopy (TEM) methods.

### MIC of nanoparticles

The MIC of AgNPs and ZnONPs for ESBLs and carbapenemase-positive isolates were done using the method described in the CLSI guideline (2018). For this test, 100 ul of MHB was added to the 12 to 1 well. Then 100 ul of nanoparticle stock solution (125 ug/mL for AgNPs and 1000 ug/mL for ZnONPs) was poured into well 12 and serial dilution was prepared from well 12 to 3. Then, 5 ul of bacteria whit a concentration of 10^7^ CFU/mL were added to all the wells except the negative control well. Finally, the microplate was incubated at 37 ^C^ for 24 h. The first dilution in which no growth was observed was considered MIC [15].

#### Minimum bactericidal concentration (MBC) of nanoparticles

After the MIC determination for each nanoparticle, an amount of 50 ul from all the wells which showed no visible bacterial growth were inoculated on MH agar plates and incubated for 24 h at 37 ^C^. When 99.9% of the bacterial population is killed at the lowest concentration of a nanoparticle agent, it is called an MBC endpoint [15].

### Cytotoxicity assay/ cell viability (MTT assay)

The cytotoxicity of AgNPs and ZnONPs was evaluated in a primary culture of fibroblast L929 cells after 24, 48, and 72 h of incubation by the MTT assay. Briefly, the cells were seeded (10^4^ cells/well) into 96-well microplates and maintained in an incubator for 24 h at 37 °C in a 5% CO2 atmosphere, then cells were treated with AgNPs and ZnONPs in DMEM in different concentrations (15.6 and 7.8 µg/ml) for AgNPs and (62.5 and 31.25 µg/ml) for ZnONPs, then incubated at 37 °C for 24, 48 and 72 h and the untreated cells served as control. Then the samples were washed with PBS (PH 7.4) and again incubated for 4 h with 100 µL/well MTT (0.5 mg/ml) to make formazan crystals. After that dissolved formazan crystals were by adding 100 µL/well DMSO. The absorbance was read with a spectrophotometer using a 570 nm filter. All experimental groups were done in five replicates. Cell viability was calculated using the following formula [16]:

$${\text{Percentage of cell viability\% = }}\frac{{sampleabsorbance}}{{controlabsorbance}} \times 100$$

### Statistical analysis

Statistical analysis was performed using SPSS version 26. The chi-square test was used to calculate statistical significance (p < 0.05).

## Results

### Study population

A total of 100 isolates of *P. mirabilis* were isolated from patients. The average age of the patients was 35.4 years (range 1 to 71 years) and the outpatients/inpatients ratio was 46/54. In general, the age group of 21–40 years had the highest frequency and the male/female ratio was 43/77.

### Detection of ESBLs and carbapenemase

In our study, 25 (25%) isolates were positive for ESBLs and 7 (7%) isolates were positive for carbapenemase enzymes. Most carbapenemase positives were able to produce ESBL (4 of 7 isolates). Among ESBLs and carbapenemase positive, 4 isolates were common between the two groups.

### Characterization of nanoparticles

**Fig. 1:**
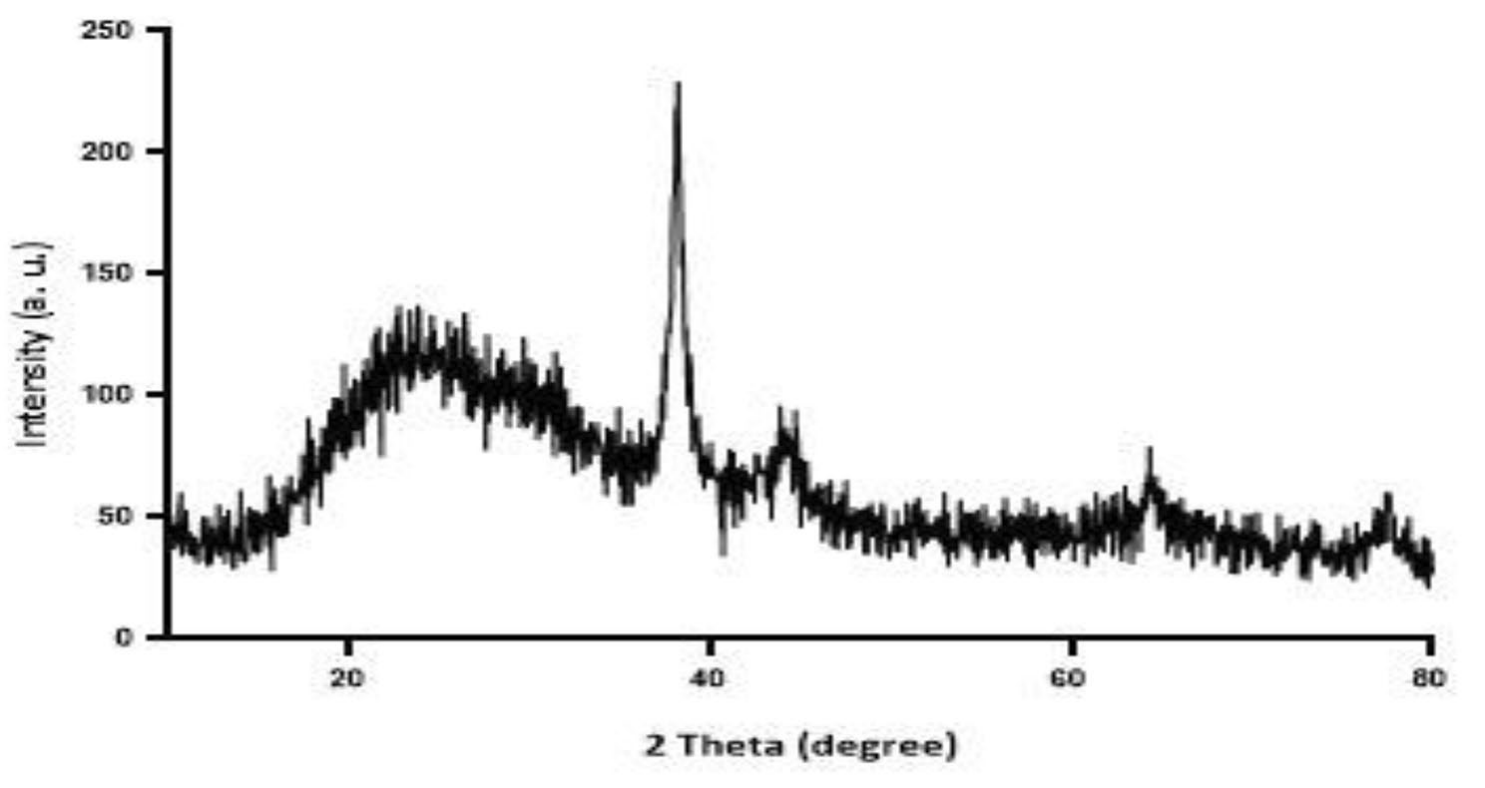
XRD diffraction pattern of synthesized AgNPs

**Fig. 2:**
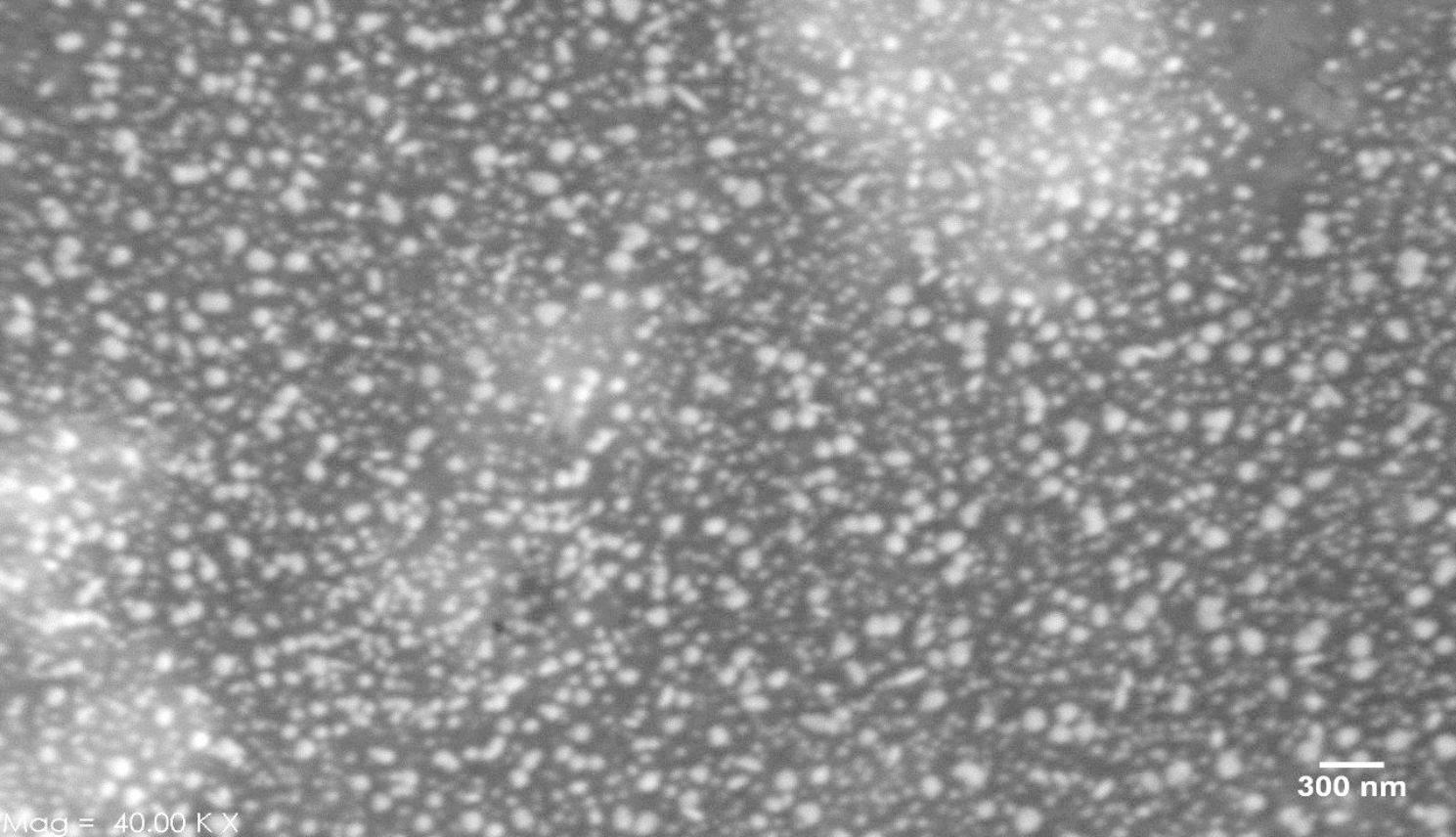
SEM images of the synthesized AgNPs with scales 300 nm

The UV-VIS absorption spectra of the AgNPs were monitored in a range of 300–500 nm. A strong peak specific for the synthesis of AgNPs was obtained at 410 nm which was specific to silver nanoparticles. The crystalline nature of the AgNPs was performed by XRD. The XRD patterns for AgNPs indicated that three chief characteristic diffraction peaks for AgNPs were observed at 2” = 38, 44, and 63 (Fig. 1). The Characterization of AgNPs was confirmed using SEM (X13, Philips, America Leo 1430vp) that the average size of the nanoparticles is about 48 nm and they are spherical (Fig. 2). In this study, ZnONPs with spherical shape and less than 70 nm size used.

### MIC and MBC of Nanoparticles

MIC and MBC testing was performed on 28 isolates. The MIC and the MBC of the ZnONPs were in the range of 31.25 µg/ml and 62.5 µg/mL, respectively. Also, for AgNPs, the MIC and the MBC were in the range of 7.8 µg/mL and 15.6 µg/mL, respectively. In the present study, MBC for AgNPs and ZnONPs was the same concentration that was obtained for the MIC. The MIC and MBC showed that *P. mirabilis* is more sensitive to AgNPs than to ZnONPs.

### Cytotoxicity assay/ cell viability (MTT assay)

Cytotoxicity assays were performed at 24, 48, and 72 h after exposure to nanoparticles by evaluating the viability of L929 cells subjected to AgNPs and ZnONPs using the MTT assay. As shown in Fig. 1, cell viability of all treatment groups with two AgNPs concentrations exceeded at least 91% of that of the control in 72 h after the incubation. So even after 72 h, no significant decrease in cell viability was observed in cells treated using AgNPs; which indicated biocompatibility and low cytotoxicity of AgNPs. Whereas the exposure of L929 cells to 62.5 µg/mL and 31.25 µg/mL ZnONPs indicated significant cytotoxicity after 24 and 72 h incubation time.

**Fig. 3:**
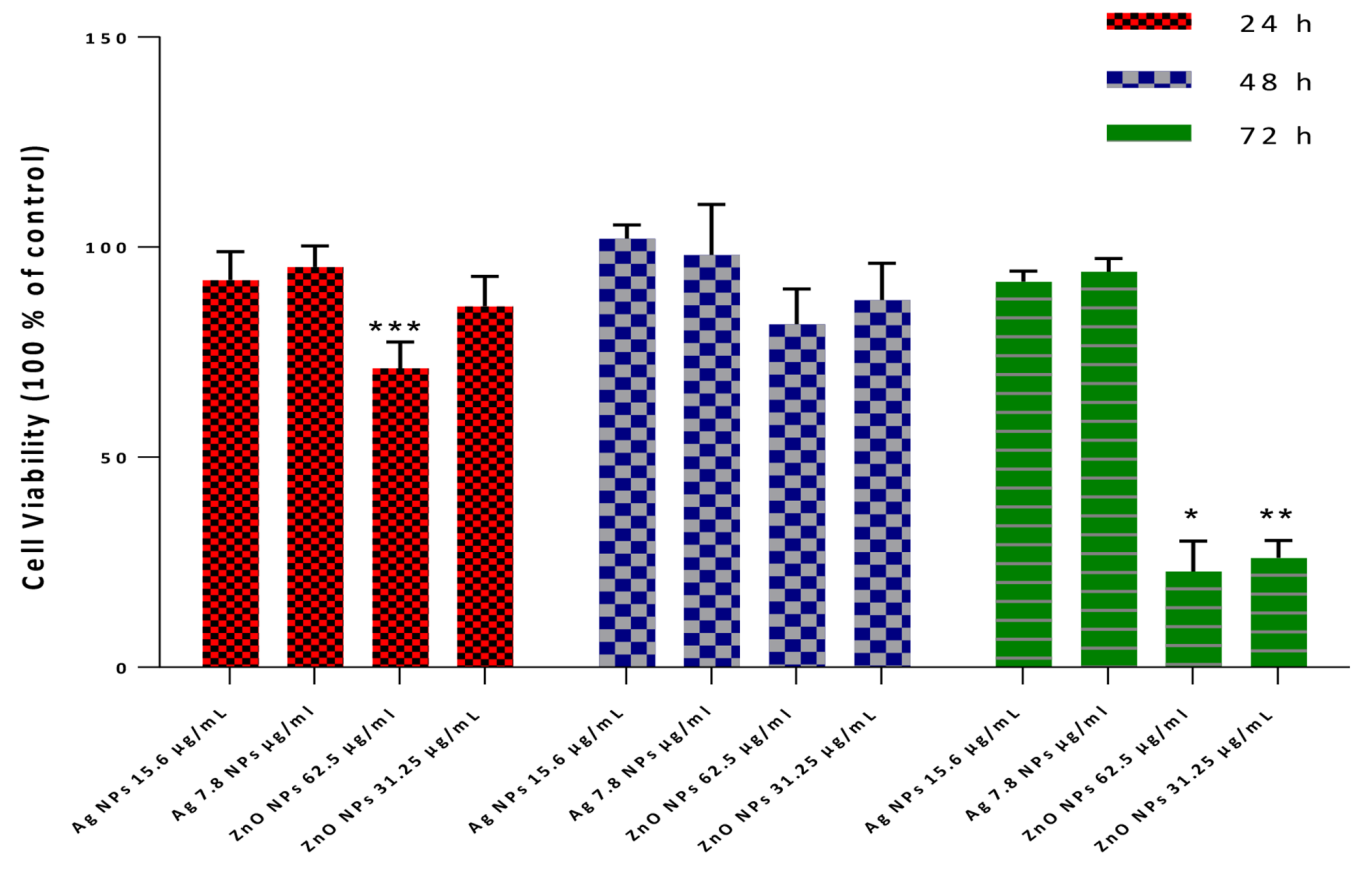
Cytotoxicity of AgNPs and ZnONPs on the viability of L929 fibroblast cells. (*P < 0.05, **P < 0.01, and ***P < 0.001 compared to untreated cells (control))

All the data were expressed as mean value ± standard deviation (SD) for five samples (n = 5). Furthermore, for ZnONPs, cytotoxicity at 15.6 µg/mL (lower than MIC) was not observed. Also, in AgNPs cytotoxicity at 62.5 µg/mL (higher than MIC) was observed (Fig. 3).

## Discussion

Infections caused by ESBLs and carbapenemase bacteria are one of the most important problems in patients [17]. In previous reports similar to the results of our study, the prevalence of ESBLs and carbapenemase-positive isolates in inpatients was significantly higher than in outpatients which can be caused by the selective pressure of using more and more diverse antibiotics in the hospitals [18, 19]. In our study, similar to the study of Mirzaei et al., the prevalence of ESBLs in *Proteus mirabilis* is significant [3]. In the study conducted by Ahmed et al., the prevalence of carbapenemase enzymes was higher than in our study [20]. According to the results of our study, the antibacterial activity of AgNPs was greater than that of ZnONPs. By our results, Elm et al. showed that the antibacterial activity of silver in gram-negative clinical isolates is greater than that of ZnO [21]. In the study of Raheem et al. in 2018, the MIC and MBC of AgNPs in one isolate of *Proteus mirabilis* have reported as 16 µg/mL and 65 ug/ml respectively which, like Parveen et al. study, is different from our study. This can be due to reasons such as the number of investigated isolates, the method of nanoparticle synthesis, the laboratory conditions of synthesis, and the characteristics of nanoparticles [22, 23]. Similar to the present study, disaanayake et al.‘ and Raheem et al. have shown that MIC and MBC were the same [22, 24].

Furthermore, in the present study, MTT assay indicated biocompatibility and low cytotoxicity of AgNPs and for ZnONPs indicated significant cytotoxicity. According to the study, the toxicity of ZnONPs can be attributed to the expression of endoplasmic reticulum (ER) stress genes, intracellular ROS (reactive oxygen species), and Zn ions [16]. Based on the results of the present study, the MIC concentration of AgNPs can be effective against *P. mirabilis* isolates isolated from clinical samples without cytotoxicity to human cells. But for ZnONPs, the MIC concentration only can be used to sterilize the environment, surfaces, and medical devices. It can also be used locally (externally). Although the role of antibiotics is significant in the treatment of bacterial infections, widespread resistance has become a warning sign to replace them. In this study, it was found that silver is more effective than ZnONPs on clinical strains of *P. mirabilis* and a concentration of AgNPs that can kill bacteria can be used as an antibacterial drug without cytotoxicity for human cells.

### Limitations

The lack of investigation on others NPs (such as copper, iron, gold, titanium, etc.) in *P. mirabilis* isolates can be mentioned as one of the chief limitations of the current study.

## Data Availability

The datasets used and/or analyzed during the current study are available from. the corresponding author on reasonable request.

## Abbreviations

ESBL: Extended- Spectrum β-lactamase
MIC: Minimum Inhibitory Concentration MBC:Minimum Bactericidal Concentration
Ag: Silver
ZnO: Zinc Oxide
NPs: Nanoparticles

## References

[1] Armbruster CE, Mobley HL, Pearson MM. Pathogenesis of *Proteus mirabilis* infection. EcoSal Plus. 2018; 8; 8(1). 10.1128/ecosalplus.esp-0009-2017 PMC5880328 29424333

[2] Adamus-Bialek W, Zajac E, Parniewski P, Kaca W (2013). Comparison of antibiotic resistance patterns in collections of *Escherichia coli* and *Proteus mirabilis* uropathogenic strains. Mol Biol Rep.

[3] Mirzaei A, Habibi M, Bouzari S, Karam MR (2019). Characterization of antibiotic-susceptibility patterns, virulence factor profiles and clonal relatedness in *Proteus mirabilis* isolates from patients with urinary tract infection in Iran. Infect drug Resist.

[4] Munita JM, Arias CA (2016). Mechanisms of antibiotic resistance. Microb spect.

[5] Talebi Bezmin Abadi A, Rizvanov AA, Haertlé T, Blatt NL (2019). World Health Organization report: the current crisis of antibiotic resistance. Bio Nano Sci.

[6] Li X, Robinson SM, Gupta A, Saha K, Jiang Z, Moyano DF, Sahar A, Riley MA, Rotello VM. Functional gold nanoparticles as potent antimicrobial agents against multi-drug-resistant bacteria. ACS Nano. 2014; 28;8(10):10682-6. 10.1021/nn5042625 PMC4212784 25232643

[7] Kasraei S, Sami L, Hendi S, AliKhani MY, Rezaei-Soufi L, Khamverdi Z. Antibacterial properties of composite resins incorporating silver and zinc oxide nanoparticles on *Streptococcus mutans* and *Lactobacillus*. Restor Dent Endod. 2014; 1; 39(2):109 – 14. 10.5395/rde.2014.39.2.109 PMC3978100 24790923

[8] Wang L, Hu C, Shao L (2017). The antimicrobial activity of nanoparticles: present situation and prospects for the future. Int Nanomed.

[9] Talebian N, Amininezhad SM, Doudi M (2013). Controllable synthesis of ZnO nanoparticles and their morphology-dependent antibacterial and optical properties. J Photochem Photobiol B: Biol.

[10] Zhang W, Niu Z, Yin K, Liu P, Chen L. Quick identification and quantification of *Proteus mirabilis* by polymerase chain reaction (PCR) assays. Annals Microbiol. 2013 Jun; 63(2):683–9.

[11] Morrissey I, Bouchillon SK, Hackel M, Biedenbach DJ, Hawser S, Hoban D, Badal RE (2014). Evaluation of the Clinical and Laboratory Standards Institute phenotypic confirmatory test to detect the presence of extended-spectrum β-lactamases from 4005 *Escherichia coli, Klebsiella oxytoca, Klebsiella pneumoniae*, and *Proteus mirabilis* isolates. J med Microbiol.

[12] Petit M, Caméléna F, Cointe A, Poncin T, Merimèche M, Bonacorsi S, Birgy A, Berçot B (2020). Rapid detection and characterization of carbapenemases in Enterobacterales with a new modified carbapenem inactivation method, mCIMplus. J Clin Microbiol.

[13] Qin Y, Ji X, Jing J, Liu H, Wu H, Yang W. Size control over spherical silver nanoparticles by ascorbic acid reduction. Colloids and Surfaces A: Physicochem Eng Asp. 2010; 3;372(1–3):172-6.

[14] Zaki S, El-Kady MF, Abd-El-Haleem D. Biosynthesis and structural characterization of silver nanoparticles from bacterial isolates. Mater res bull. 2011; 1;46(10):1571-6.

[15] Parvekar P, Palaskar J, Metgud S, Maria R, Dutta S (2020). The minimum inhibitory concentration (MIC) and minimum bactericidal concentration (MBC) of silver nanoparticles against *Staphylococcus aureus*. Biomater Investig Dent.

[16] Wang M (2018). A comparative study of toxicity of TiO2, ZnO, and Ag nanoparticles to human aortic smooth-muscle cells. Int J Nanomedicine.

[17] Moura ML, Boszczowski I, Blaque M, Mussarelli RM, Fossaluza V, Pierrotti LC, Campana G, Brandileone MC, Zanella R, Almeida SC, Levin AS (2022). Effect on Antimicrobial Resistance of a policy restricting Over-the-Counter Antimicrobial sales in a large Metropolitan Area, São Paulo, Brazil. Emerg infects dis.

[18] Gajdács M, Urbán E. Comparative epidemiology and resistance trends of proteae in urinary tract infections of inpatients and outpatients: a 10-year retrospective study. Antibiotics. 2019; 11;8(3):91. 10.3390/antibiotics8030091 PMC6783862 31373311

[19] Wang JT, Chen PC, Chang SC, Shiau YR, Wang HY, Lai JF, Huang IW, Tan MC, Lauderdale TL (2014). Antimicrobial susceptibilities of *Proteus mirabilis*: a longitudinal nationwide study from the Taiwan surveillance of antimicrobial resistance (TSAR) program. BMC infects dis.

[20] Ahmed DA, Hasan NM. Phenotypic detection of some Beta-lactamases in local isolates of *Proteus mirabilis*.2022.

[21] Saleh ELMGM (2017). Investigation of the efficacy of synthesized silver and zinc oxide nanoparticles against multi-drug resistant gram negative bacterial clinical isolates. Arch Clin Microbiol.

[22] Raheem HQ, Al-Thahab A, Abd FG (2018). Antibacterial activity of silver nanoparticles extracted from *Proteus mirabilis* and healing the wound in rabbit. Biochem Cell Arch.

[23] Parveen A, Yalagatti MS. Venkataraman Abbaraju, and Raghunandan Deshpande. “Emphasized mechanistic antimicrobial study of biofunctionalized silver nanoparticles on model *Proteus mirabilis*.“ J drug deliv. 2018; 10(1). 10.1155/2018/3850139 PMC5987338 29951316

[24] Disaanayake DM, Faoagali J, Laroo H, Hancock G, Whitehouse M (2014). Efficacy of some colloidal silver preparations and silver salts against *Proteus* bacteria, one possible cause of rheumatoid arthritis. Inflammopharmacology.

